# Plant Species Diversity Improves Soil Physicochemical Traits and Modulates Soil Microbial Community Structure, with a Pronounced Enhancement of Fungal Diversity in Urban Forests

**DOI:** 10.3390/plants15010079

**Published:** 2025-12-26

**Authors:** Yu-Hang Song, Fan-Bing Xu, Ming-Hui Wang, Yuan-Bo Xie, Li-Ming Tian, Cai-Xia Lv, Xi-Wen Zhang, Zi-Ming Guo, Dan Zhang

**Affiliations:** College of Landscape Architecture, Changchun University, Changchun 130022, China; 231201437@mails.ccu.edu.cn (Y.-H.S.); 241201420@mails.ccu.edu.cn (F.-B.X.); 241203452@mails.ccu.edu.cn (M.-H.W.); 241202441@mails.ccu.edu.cn (Y.-B.X.); 251203475@mails.ccu.edu.cn (L.-M.T.); 251203461@mails.ccu.edu.cn (C.-X.L.); 251203479@mails.ccu.edu.cn (X.-W.Z.); 251203476@mails.ccu.edu.cn (Z.-M.G.)

**Keywords:** species diversity, soil microbial community, functional gene, urban forest, nutrient cycling

## Abstract

Urban parks are key to urban ecosystems, where soil microbe-plant-soil interactions sustain ecosystem services. Using high-throughput sequencing and multivariate statistics, this study explored how plant species diversity affects soil microbial community structure, functional diversity, and environmental drivers. Results showed that fungal and bacterial OTUs differed across plant diversity gradients, with Ascomycota (fungi) and Actinobacteriota/Proteobacteria (bacteria) dominant. Soil organic carbon (SOC) was positively correlated with Verrucomicrobia, while Acidobacteriota increased with lower SOC. Fungi were more sensitive to pH than bacteria. Partial Least Squares Path Modeling (PLS-PM) indicated that plant diversity was significantly positively associated with fungal community structure and was indirectly associated with bacterial diversity via soil factors (e.g., SOC, pH), with fungal community variation more explained than bacterial. Higher plant diversity was associated with elevated SOC and a higher relative abundance of putative nutrient-cycling taxa (e.g., *Rhizobium*), suggesting a potential enhancement of soil nutrient cycling capacity. This study demonstrates that plant diversity shapes microbial communities directly and via soil properties, highlighting synergistic effects. We propose arbor-shrub-herb composite vegetation in urban forest management to optimize microbial habitats and ecological services.

## 1. Introduction

Urban forest parks, as pivotal spatial carriers of urban ecosystems, play irreplaceable roles in preserving urban biodiversity and safeguarding the stability of ecosystems [1,2]. Underground ecosystems sustain complex, dynamically balanced microbial community networks that are closely linked to aboveground plant communities and ecosystem functioning [3,4]. With the advancement of urban ecological research, microorganisms—recognized as core drivers of ecosystem material cycling, key participants in plant symbiotic relationships [5,6], and primary agents of organic matter decomposition [7]—have emerged as novel biological indicators for evaluating the health of urban ecosystems [8,9].

Plant community composition regulates soil traits via direct pathways, including litter deposition and root exudate secretion [10,11]. Bardgett R.D. et al. [6] reported that plant litter—a primary source of soil organic matter and nutrients—alters soil pH and soil organic carbon (SOC) during decomposition, thus indirectly restructuring the soil microenvironment. In urban forest parks, soil physicochemical properties exhibit distinct characteristics across plant species richness gradients [12,13]. Low, medium, and high diversity levels correspond to variations in litter quality/quantity and root activity, which may in turn drive changes in electrical conductivity (EC), soil water content (SWC), and total phosphorus (TP) [14,15]. These physicochemical differences establish the foundation for soil microbial community assembly. Delgado-Baquerizo M. et al. [1,16] showed that arbor-shrub-herb composite vegetation systems create differentiated microhabitats that alter soil properties and promote microbial niche differentiation—confirming that soil properties as crucial bridges linking plant species diversity and microbial communities.

Plant species diversity shapes soil microbial community structure and composition through multiple pathways, encompassing both direct and indirect drivers [4,17]. Zhou T. et al. [18] found that increased plant species richness enhances root exudate diversity and litter quality, significantly boosting soil microbial diversity, biomass, and functional metabolic activity; Lange M. et al. [19] further demonstrated that higher plant species diversity promotes fungal diversity, network complexity, and stability. Direct drivers of soil microbial community assembly include plant community composition, root exudates, and litter characteristics—plant community composition determines the types and proportions of resource inputs, the chemical diversity of root exudates provides metabolic substrates for microorganisms, and heterogeneity in litter decomposition shapes microbial microenvironments while directly regulating microbial species sorting and functional differentiation [10,20,21]. In urban forest parks, microbial community structures vary markedly across plant species diversity gradients: low diversity may constrain microbial development due to resource limitation, whereas medium and high diversity support the coexistence of more bacteria and fungi through abundant resources and complex microhabitats [14,15,22], and Shen C. et al. [4] further confirmed that high-diversity communities increase the complexity of soil organic matter and activate specialized functional microbial groups. Indirectly, soil physicochemical properties act as key intermediaries linking plants and microorganisms; Jiang et al. [23] demonstrated that plant species diversity under grazing disturbance indirectly shapes microbial niche differentiation by altering soil properties such as total nitrogen, organic carbon, and pH, with high-diversity communities, for instance, elevating surface soil organic carbon (SOC) content to provide richer carbon sources for fungi and thereby enhance litter decomposition. However, urban plant-microbe interactions differ fundamentally from natural ecosystems due to anthropogenic disturbances (e.g., soil compaction, urban heat islands), and many understudied urban subtypes (e.g., cold-temperate urban forests) lack targeted investigations [24,25]. A recent global review by Monaco et al. [24] systematically highlighted that root exudate-mediated microbial assembly dominates soil nutrient cycling in urban habitats, while cold-temperate urban forests—characterized by seasonal freeze-thaw cycles—have critical knowledge gaps in plant-microbe feedback regulation. This research deficiency motivated our focus on Jingyuetan National Forest Park, a representative cold-temperate urban forest, to clarify microbial community dynamics across plant diversity gradients.

Jingyuetan National Forest Park in Changchun City, a representative urban forest park in Northeast China, is characterized by high forest coverage, strong ecosystem integrity, and diverse vegetation types [26]. Long-term ecological succession has formed a stratified and complex vegetation ecosystem, which fosters considerable plant species diversity. Taking Jingyuetan National Forest Park as the research area, this study systematically analyzes the structural characteristics and diversity patterns of soil microbial communities across plant species diversity gradients, as well as the correlations between environmental factors and microbial communities. The findings of this study aim to provide a theoretical basis and data support for urban forest health assessment, scientific management, and biodiversity conservation.

## 2. Results

### 2.1. Soil Physicochemical Properties Among Different Levels of Plant Species Diversity

**Figure 1:**
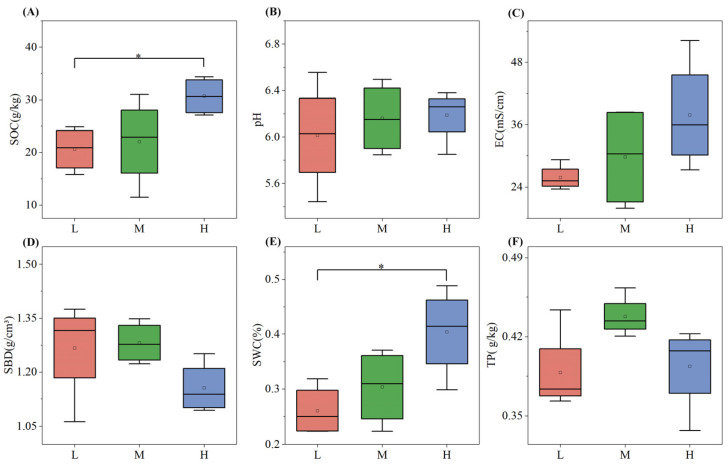
Soil SOC (**A**), pH (**B**), EC (**C**), SBD (**D**), SWC (**E**), and TP (**F**) under different plant species diversity levels (L = low, M = medium, H = high).In the boxplots: Whiskers represent 1.5 × interquartile range (1.5 × IQR); the line inside the box denotes the Median; the dot indicates the Mean. Intergroup differences were analyzed via one-way ANOVA followed by LSD multiple comparisons; * *p* < 0.05 indicate significant differences.

Soil physicochemical properties varied significantly across different plant species diversity levels (Figure 1). With the increase in plant species diversity, the values of SOC, SWC, and EC increased. Notably, SOC in the high plant species diversity group was significantly higher than that in the low plant species diversity group by about 48.4% (*p* < 0.05), and SWC in the high group was significantly higher than that in the low group by approximately 53.8% (*p* < 0.05). pH and EC increased gradually with the increase in plant species diversity, yet the differences were not significant. In contrast, soil bulk density (SBD) showed an opposite trend, decreasing as plant species diversity increased. Particularly, TP was highest under medium plant species diversity.

### 2.2. Effects of Plant Species Diversity on Soil Microbial Community Composition and Diversity

#### 2.2.1. Effects of Plant Species Diversity on Soil Microbial OTU Composition

**Figure 2:**
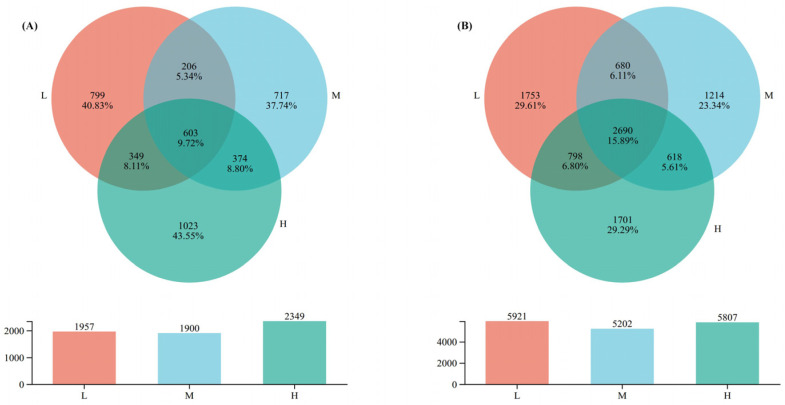
Number of soil microbial OTUs. (**A**) Fungi under different plant species diversity levels, (**B**) Bacteria under different plant species diversity levels. The numerical display of the bar chart shows that the number of OTUs under medium plant species diversity is in an intermediate position compared with low and high levels.

The VENN diagram (Figure 2) was used to visually display the similarities and differences in OTU composition under different levels of plant species diversity. In the fungal community, the total number of OTUs under high plant species diversity was 2349, higher than the low and the medium plant species diversity levels, with values of 1957 and 1900, respectively. Moreover, unique OTUs under high plant species diversity reached 1023, accounting for 43.55% of the total OTUs in the high plant species diversity fungal community. However, in the bacterial community, the total number of OTUs under low plant species diversity was 5921, with unique OTUs of 1753 accounting for 29.61% of the total OTUs in the low plant species diversity bacterial community, which was higher than those under medium (5202) and high (5807) plant species diversity levels.

#### 2.2.2. Soil Microbial Community Composition and Functional Potential Characteristics Under Different Levels of Plant Species Diversity

At the fungal phylum level, Basidiomycota had the highest relative abundance under the three plant species diversity levels (accounting for 42.32% of the total OTUs), followed by Ascomycota (39.34%) and Mortierellomycota (8.33%). Under high plant species diversity, the relative abundance of Basidiomycota was the lowest, and the relative abundance of Ascomycota was the highest; Basidiomycota had the highest relative abundance under medium plant species diversity.

**Figure 3:**
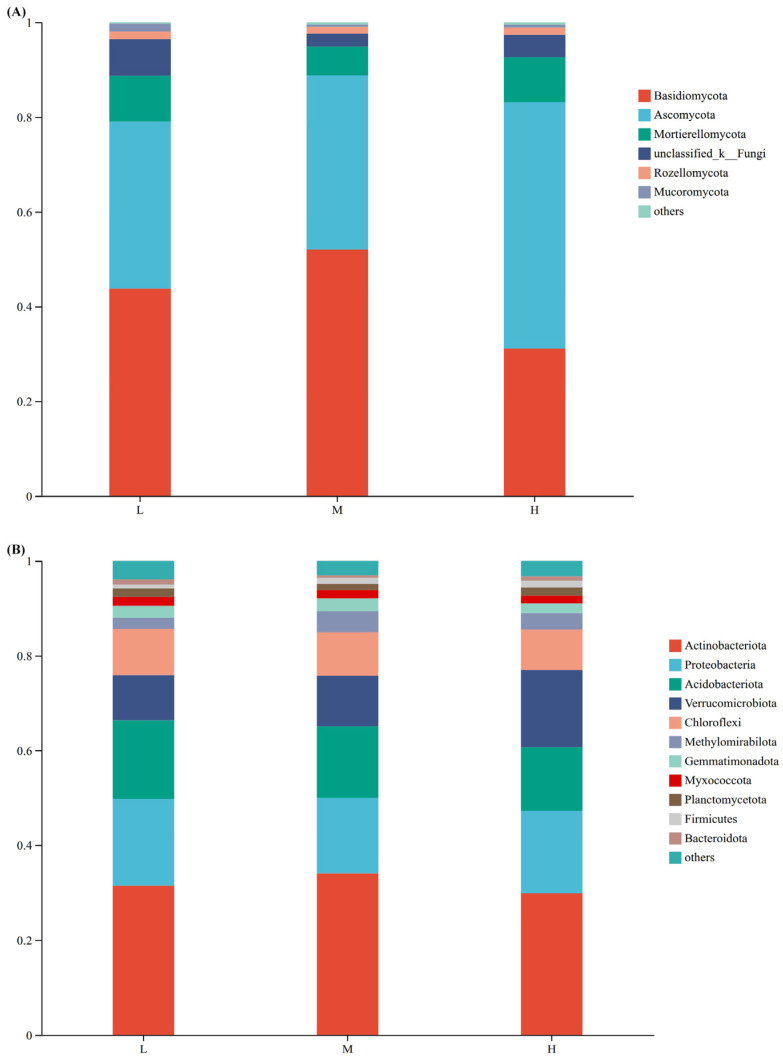
Relative abundances of soil fungi (**A**) and bacteria (**B**) at the phylum level under different plant species diversity levels.

At the phylum level of bacteria (Figure 3B), the community composition differed among different plant species diversity levels, though the differences were not significant. The top five dominant bacterial phyla, in descending order of relative abundance, were Actinobacteriota, Proteobacteria, Acidobacteriota, Verrucomicrobiota, and Chloroflexi. Among them, Actinobacteriota had the highest proportion across all levels: under low plant species diversity, it accounted for approximately 31%, under medium diversity around 34%, and under high diversity about 29%.

**Figure 4:**
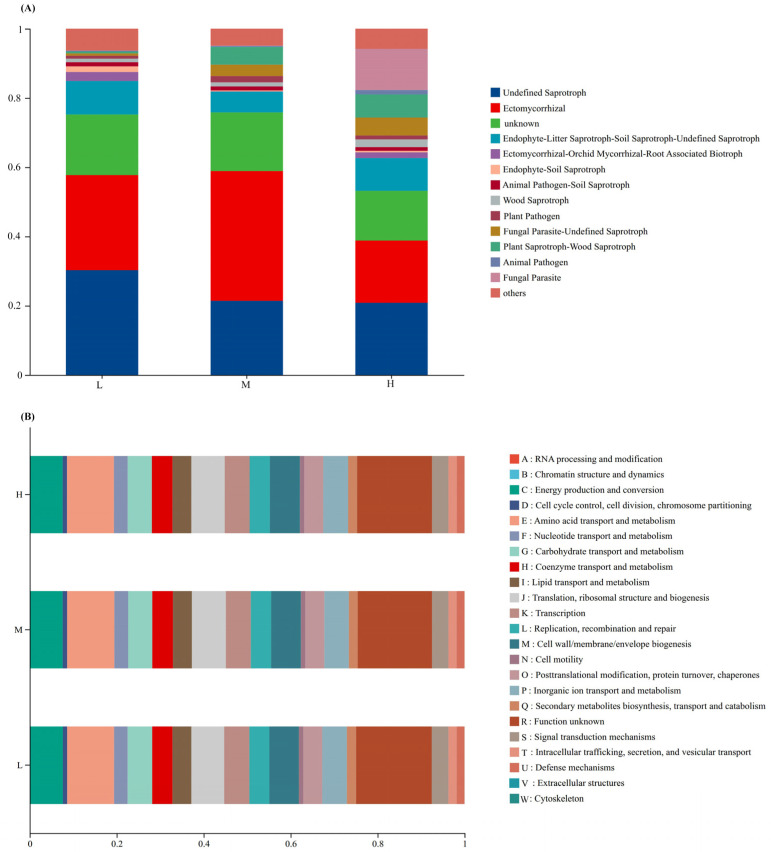
Functional group classification of fungi by FUNGuild (**A**) and COG functional classification (**B**) of bacteria under different plant species diversities.

FUNGuild (Fungal Functional Guild) was used to obtain the functional classification of fungi in samples and the abundance information of each functional classification under different plant species diversity levels (Figure 4A). With the increase of plant species diversity, the relative abundances of Plant Saprotroph-Wood Saprotroph and Fungal Parasite-Undefined Saprotroph increased. However, the relative abundance of Ectomycorrhizal was the highest under medium plant species diversity. In addition, Fungal Parasite was only detected under high plant species diversity, and these fungal parasites can infect and potentially control the population sizes of their host organisms, further mediating biotic interactions within the ecosystem.

Clusters of Orthologous Groups of proteins (COG) functional annotation of OTUs was performed to obtain OTU annotation information at the COG functional level and the abundance information of each function in different samples (Figure 4B). The types and abundances of bacterial functions showed little change under different plant species diversity levels.

It should be noted that fungal functional annotations were derived from sequence homology matching via the FUNGuild database, and bacterial functional classifications were based on macro-pathway assignments from the COG database. Both approaches only reflect the potential functional roles of microbial taxa and cannot verify in-situ functional activity (e.g., the symbiotic efficiency of ectomycorrhizal fungi or the in-vivo expression intensity of metabolic pathways). Confirmation of actual microbial functions requires further validation via metatranscriptomics, proteomics, or enzyme activity assays.

#### 2.2.3. Effects of Plant Species Diversity on Soil Microbial Community Diversity Indices

**Figure 5:**
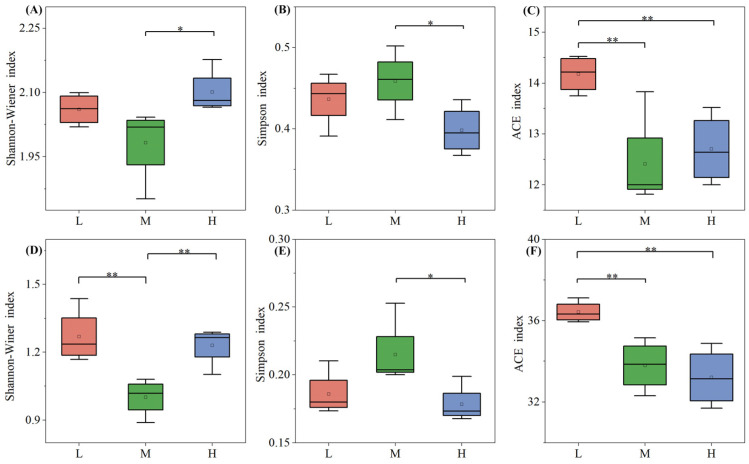
Alpha diversity indices of fungi (**A**–**C**) and bacteria (**D**–**F**) under different plant species diversity levels. In the boxplots: Whiskers represent 1.5 × interquartile range (1.5 × IQR); the line inside the box denotes the Median; the dot indicates the Mean. Indices were analyzed via one-way ANOVA with LSD multiple comparisons; * *p* < 0.05, ** *p* < 0.01 denote significant intergroup differences.

The soil fungal and bacterial community Alpha diversity indices across different plant species diversity levels (Figure 5) reveal a distinct pattern: the Shannon-Wiener index—one of the most representative Alpha diversity metrics—was lowest at the medium level. This trend was consistent in both microbial communities, indicating a non-linear response of microbial diversity to plant species diversity changes. For the soil microbial Simpson index, the Shannon-Wiener index and Ace richness index of soil microorganisms under high plant species diversity were significantly different from those under low and medium levels (*p* < 0.05). In the ACE indices of soil fungi and bacteria, the low-level plant species diversity index was the highest and significantly different from the medium and high levels (*p* < 0.01). Notably, for the bacterial ACE index, it showed a significant decreasing trend as the plant species diversity level increased, with the highest values observed under low plant diversity.

### 2.3. Significance Tests for Soil Bacteria and Fungi Under Different Levels of Plant Species Diversity

**Figure 6:**
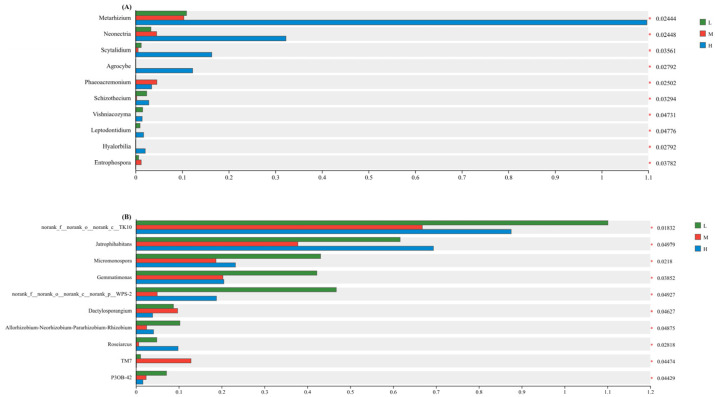
Intergroup difference significance tests of soil fungi (**A**) and bacteria (**B**) at the genus level (* *p* < 0.05).

The intergroup difference significance test for dominant microbial genera (Figure 6) was conducted using Linear Discriminant Analysis Effect Size (LEfSe) with an LDA threshold of 2.0 and *p*-value cutoff of 0.05. LEfSe was selected for its ability to integrate statistical significance with biological relevance, prioritizing taxa that contribute most to community differentiation across plant diversity gradients [27]. The significance test of intergroup differences in dominant genera (Figure 6) of soil fungi and bacteria under different levels of plant species diversity showed that 10 dominant fungal genera and 10 dominant bacterial genera all had significant differences under low, medium, and high plant species diversity (*p* < 0.05). At the fungal genus level, the relative abundances of *Metarhizium*, *Neonectria*, *Scytalidium*, and *Agrocybe* under high plant species diversity were significantly higher than those under low and medium levels. At the bacterial genus level, the relative abundances of *Jatrophihabitans*, *Micromonospora*, *Gemmatimonadetes*, and *Allorhizobium-Neorhizobium-Pararhizobium-Rhizobium* under medium plant species diversity were significantly lower than those under low and high levels.

### 2.4. Interaction Analysis of Soil Bacteria, Fungi, and Soil Physicochemical Properties

#### 2.4.1. Correlation Heatmap Analysis

**Figure 7:**
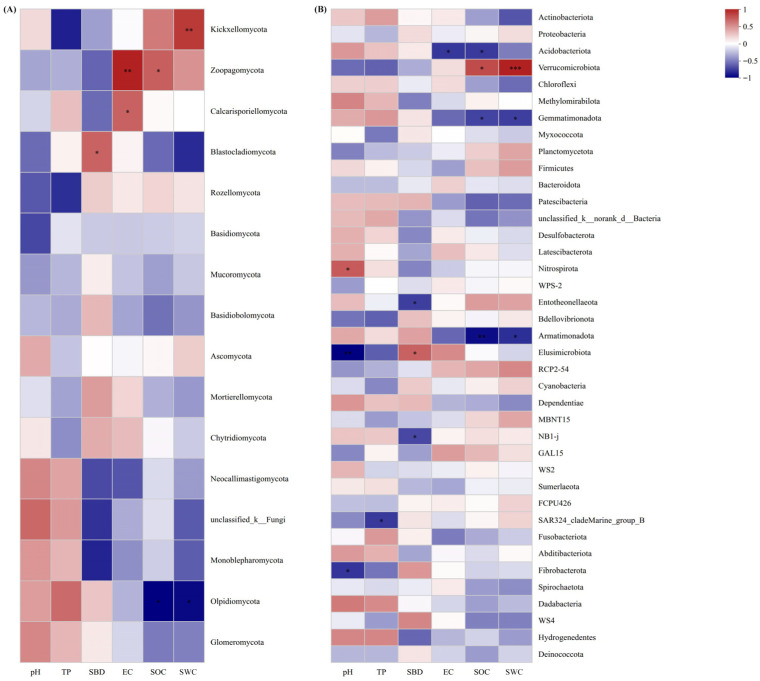
Relationships between soil physicochemical properties and fungal (**A**) and bacterial (**B**) community compositions. (* *p* < 0.05, ** *p* < 0.01, *** *p* < 0.001).

At the fungal phylum level (Figure 7A), the OTU number of Blastocladiomycota was negatively correlated with SBD (*p* < 0.05); Zoopagomycota OTU number was significantly negatively correlated with EC (*p* < 0.01); Kickxellomycota OTU number was negatively correlated with SWC; Olpidiomycota OTU number was significantly positively correlated with SOC and SWC (*p* < 0.01). For bacteria (Figure 7B), SOC was significantly negatively correlated with the OTU numbers of Armatimonadota, Acidobacteriota, and Gemmatimonadetes (*p* < 0.05, *p* < 0.01), but positively correlated with Verrucomicrobia OTU number (*p* < 0.05). SWC was negatively correlated with Armatimonadota and Gemmatimonadetes OTU numbers (*p* < 0.05), and extremely significantly positively correlated with Verrucomicrobia OTU number (*p* < 0.001).

#### 2.4.2. Mantel Test Heatmap Analysis

**Figure 8:**
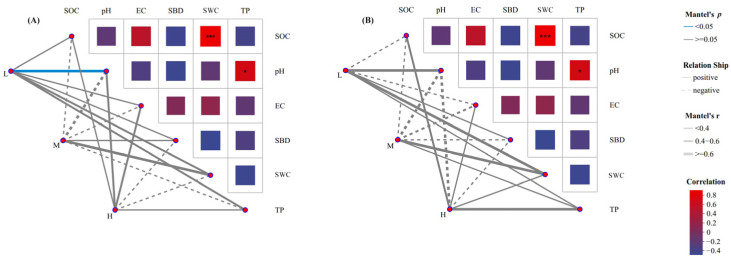
Mantel test analysis of soil physicochemical properties and fungal (**A**) and bacterial (**B**) community compositions at the phylum level under different plant species diversity levels. (* *p* < 0.05, *** *p* < 0.001).

Mantel test analysis was performed on the relative abundances of soil microorganisms and soil physicochemical factors at different plant species diversity levels (Figure 8). The results showed that in the fungal community, soil pH was significantly correlated with low plant species diversity (*p* < 0.05). The compositions of soil fungal and bacterial communities under different plant species diversity levels had significant relationships with soil physicochemical properties, among which SOC, SWC, pH, and TP were important influencing factors for the compositional differences of soil fungal and bacterial communities under different plant species diversity levels.

#### 2.4.3. PLS-PM Analysis

**Figure 9:**
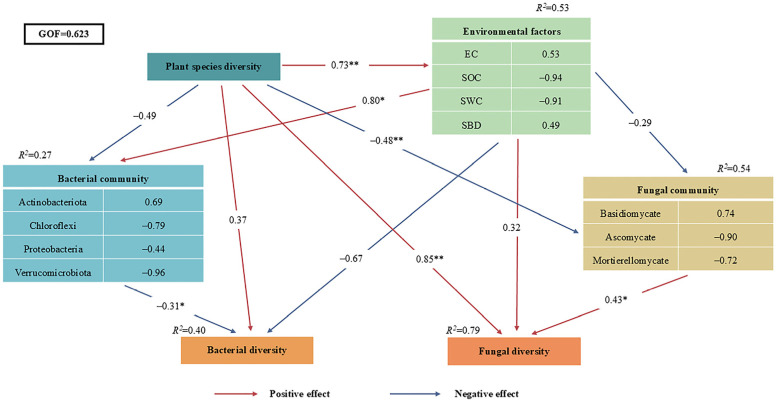
Partial least squares path modeling (PLS-PM) of different plant species diversity levels and soil properties. The red and blue arrows represent positive and negative relationships, respectively. Adjacent numbers labeled in the same direction as the arrow represent standardized path coefficients. Values of *R*^2^ indicate the proportion of variance explained by each variable in the model. (* *p* < 0.05, ** *p* < 0.01,).

PLS-PM analysis further explored the functional relationships among different levels of plant species diversity, soil physicochemical properties, soil bacterial and fungal diversity indices, and soil bacterial and fungal community structures (Figure 9). The model accounted for 53% of the variance in Environmental factors, and explained 27%, 54%, 40%, and 79% of the variance in Bacterial community, Fungal community, Bacterial diversity, and Fungal diversity, respectively. The results demonstrated that species diversity exerted a significant direct association with environmental factors (*λ* = 0.73, *p* < 0.01) and bacterial diversity (*λ* = −0.48, *p* < 0.01). Meanwhile, plant species diversity was significantly positively associated with fungal community structure, and the PLS-PM model suggested a potential pathway whereby plant diversity indirectly affected bacterial diversity through soil physicochemical factors (e.g., SOC, pH). The model explained 79% of the variation in fungal communities (*R*^2^ = 0.79) and 40% of that in bacterial communities (*R*^2^ = 0.40), with fungal communities showing stronger responses to plant diversity than bacterial communities. Additionally, species diversity indirectly correlated with fungal community (*λ* = 0.85, *p* < 0.01) by modulating Environmental factors, and ultimately impacted Bacterial community (*λ* = −0.31, *p* < 0.05) and Fungal diversity (*λ* = 0.43, *p* < 0.05).

The PLS-PM model only reveals hypothesized directional associations between variables, which are consistent with ecological theory but cannot establish causal relationships due to the observational nature of the data. The inferred pathways require further validation through controlled experiments.

## 3. Discussion

### 3.1. Effects of Plant Species Diversity on Soil Properties

Soil physicochemical properties, core to ecosystem material cycling and energy flow, are modulated by plant species diversity via resource input (e.g., litter, root exudates) and habitat optimization (e.g., microclimate regulation) [14,19]. This study found that SOC and SWC in high-diversity plots were 48.4% and 53.8% higher than in low-diversity plots (*p* < 0.05), while SBD decreased significantly with increasing diversity (Figure 1)—consistent with the “resource complementarity hypothesis”, which holds that high plant diversity boosts litter/root exudate quantity/quality to enhance carbon sequestration and water retention [18].

Elevated SOC in high-diversity plots stems from two key processes: diverse litter (e.g., mixed conifer-broadleaf litter here) provides continuous heterogeneous carbon, extending microbial “substrate utilization periods” to slow soil organic matter (SOM) decomposition [11]; root exudates (e.g., phenolic acids) stimulate decomposers like Actinobacteriota, promoting stable SOM formation via the “microbial carbon pump” [28]. A global study further confirmed that each 10% increase in plant richness raises SOC by 3.2% on average, with stronger effects in temperate forests [16]. Reduced SBD with higher diversity (Figure 1D) links to enhanced root development and soil aggregation: high-diversity communities have complex root architectures (e.g., arbor-shrub-herb mixed roots) that loosen soil and increase macroporosity [29], while root exudates (e.g., polysaccharides) and microbial metabolites (e.g., mycorrhizal glomalin) stabilize aggregates [2]—consistent with urban forest observations where composite vegetation reduced SBD by 15–20% vs. single-species stands [30].

Notably, TP peaked at medium diversity (Figure 1F), reflecting balanced input and uptake: moderate litter decomposition and optimized mycorrhizal phosphorus acquisition maintain high TP [23], whereas high diversity increases plant uptake and microbial mineralization, lowering TP slightly [31]. This “intermediate effect” is universal across ecosystems like temperate deciduous forests [17]. Soil pH and EC showed non-significant increases with diversity (Figure 1B,C): temperate soil buffering offsets pH lowering from organic acid release (e.g., humic acids) via base cation accumulation (e.g., Ca^2+^, Mg^2+^) [32], while higher EC reflects increased dissolved organic matter/mineral ions from enhanced microbial activity [33].

In summary, plant diversity regulates soil properties via carbon sequestration, structure improvement, and nutrient optimization—creating favorable microbial habitats and sustaining urban forest functions (e.g., carbon storage, water regulation). Promoting plant diversity thus serves as an effective strategy for urban forest soil quality and ecological service enhancement.

### 3.2. Effects of Plant Species Diversity on Soil Microbial Community Structure

This study confirmed that increased plant species diversity significantly altered the composition of soil fungal communities. This aligns with the theory that plant species diversity drives microbial differentiation through resource complementarity and habitat heterogeneity [17]. For instance, unique fungal OTUs comprised 43.55% of communities in high-diversity plots (Figure 2A), and the relative abundance Ascomycota markedly increased (Figure 3A). These shifts likely relate to enhanced litter chemical diversity and root exudate heterogeneity in high plant species diversity plots. High diversity supports diverse litter types (e.g., coniferous needles with high lignin and broadleaves with high cellulose), creating heterogeneous carbon/nutrient inputs [19]. Variations in litter lignin: cellulose ratios and nutrient concentrations form diversified niches—Basidiomycota—renowned for secreting high-activity lignocellulolytic enzymes (e.g., laccases, lignin peroxidases)—dominate in medium-diversity plots; Ascomycota, by contrast, exhibit strong cellulolytic capacity and are enriched in high-diversity plots due to heterogeneous cellulose inputs from mixed broadleaf-conifer litter [7,34]. Ascomycota do not produce lignin-specific degrading enzymes, distinguishing their functional niche from Basidiomycota. Additionally, high diversity boosts root exudate heterogeneity (e.g., phenolic acids like vanillic acid, sugars like glucose), which selectively stimulates Ascomycota growth by upregulating their nutrient acquisition genes [21]. Specifically, root-derived phenolic acids (e.g., vanillic acid) may selectively enrich bacterial degraders like Pseudomonas via allelopathy [10], while varied litter quality provides diversified carbon sources for saprophytic fungi (e.g., Basidiomycota) [7]. Recent evidence further indicates that root exudate diversity enhances microbial network complexity [21], supporting the hypothesis that that multidimensional resource inputs drive microbial functional sorting.

While fungal communities exhibited the expected positive response to increased plant diversity, the observation that bacterial ACE richness peaked under low plant diversity (Figure 5D–F) deviates from the core tenet of the “diversity begets diversity” hypothesis—which posits that higher plant diversity boosts belowground microbial diversity via amplified resource heterogeneity and expanded niche complexity [18,19]. Unlike the consistent plant-fungal diversity correlation, this counterintuitive bacterial pattern does not imply direct causality; instead, it likely reflects the selective dominance of oligotrophic taxa (e.g., Acidobacteriota) in simplified plant communities, which are evolutionarily adapted to maintain high species richness under resource-limited conditions [17]. In our study, the relative abundance of Acidobacteriota—a representative oligotrophic phylum with recalcitrant carbon utilization capacity and nutrient scarcity tolerance [17]—was significantly higher in low-diversity plots (22.3%) than in medium- (18.7%) and high-diversity (16.2%) plots (*p* < 0.05). This taxonomic enrichment provides indirect evidence that oligotrophic bacteria drive elevated bacterial ACE richness in low-diversity habitats, as these stress-tolerant groups can colonize and diversify in niches where copiotrophic bacteria (dependent on labile organic matter from diverse plant inputs) are competitively excluded. This divergent response of fungi and bacteria to plant diversity gradients also highlights the distinct niche preferences of different microbial taxa, which in turn shape their functional contributions to soil biogeochemical cycles.

Importantly, the functional roles of key microbial groups and dominant bacterial phyla are tightly linked to P cycling and SOC dynamics observed in this study, while plant diversity also drives distinct patterns in microbial OTU uniqueness and functional group abundance. Ectomycorrhizal fungi—whose abundance peaked under medium diversity (Figure 4A)—form symbioses with dominant trees (e.g., *Pinus koraiensis*, *Quercus mongolica*), enhancing plant P acquisition via extended root nutrient uptake and acid phosphatase secretion to mineralize insoluble P [4,35]. This aligns with our finding of higher total phosphorus (TP) under medium diversity (Figure 1F), as balanced symbiosis here reduces P immobilization; it also boosts plant stress resistance to ensure stable litter input, an indirect driver of SOC accumulation [33]—a pattern supported, who noted mycorrhizal fungi maximize nutrient-exchange mutualism under intermediate resource conditions [23].

Meanwhile, Plant Saprotroph-Wood Saprotroph fungi (abundance increasing with diversity, Figure 4A) drive SOC turnover by decomposing heterogeneous litter (e.g., high-lignin coniferous needles, high-cellulose broadleaves) via lignin peroxidases [36]. Though they release labile carbon for microbial metabolism, a portion of decomposed C forms stable SOC (e.g., humic substances) via the “microbial carbon pump” [7,28]—explaining why high-diversity plots had 48.4% higher SOC than low-diversity plots (Figure 1A), as heterogeneous litter slows complete C mineralization [11,19]. Complementing fungal functions, dominant high-diversity bacterial phyla Actinobacteriota and Proteobacteria (Figure 3B) reinforce C cycling. Actinobacteriota contribute to the decomposition of labile-to-semi-recalcitrant organic matter (e.g., cellulose, hemicellulose) via cellulase-encoding genes; their activity is elevated by high SOC, which accelerates labile carbon turnover. For recalcitrant organic matter (e.g., lignin), Basidiomycota (via lignin peroxidases) and specialized Actinobacteriota taxa (via laccases) are the primary decomposers, as cellulase genes do not target lignin [37,38].

In contrast, unique bacterial OTUs were lowest in medium-diversity plots (Figure 2B)—a pattern linked to “intermediate competition effects”: moderate carbon/nutrient supplies fail to meet oligotroph needs (e.g., Acidobacteriota) or sustain copiotroph proliferation, suppressing microbial metabolic activity [39]. Globally, intermediate plant diversity disrupts soil pH and nutrient balance, inhibiting niche-specialized microorganisms [40]. Additionally, Fungal Parasite abundance increased with diversity (Figure 4A), potentially reflecting activated plant pathogen defense mechanisms that enrich parasitic taxa to regulate pathogens [41,42]. Together, these processes support the “multidimensional resource input” hypothesis, where diverse plant resources select for taxa optimizing P cycling, SOC dynamics, and microbial community assembly in urban forest soils [43].

### 3.3. Interaction Mechanisms Between Soil Physicochemical Properties and Soil Microorganisms

Beyond direct biological interactions, soil physicochemical properties (SOC, pH, SWC) mediate plant-microbe linkages. SOC positively correlated with Verrucomicrobia but negatively with Acidobacteriota (Figure 7B), likely because SOC provides stable carbon for oligotrophic Verrucomicrobia [39], while Acidobacteriota adapts to low-organic-matter environments [44]. This supports Six et al.’s [37] “organic carbon stability” theory. Notably, Verrucomicrobia functional genes (e.g., methane monooxygenase) upregulate under high SOC [45], confirming their carbon metabolic dependence.

While niche theory typically emphasizes environmental filtering of microbial communities at the genus level (where functional specificity is higher) [6], we first analyzed community patterns at the phylum level to capture broad-scale shifts in microbial trophic strategies (e.g., oligotrophic vs. copiotrophic) along plant diversity gradients. Phylum-level analysis revealed overarching trends (e.g., decreased Acidobacteriota with increasing SOC) that provided a framework for interpreting finer-scale genus-level dynamics (Figure 6), which is consistent with Prober et al. [40] who reported that plant diversity primarily shapes fungal β-diversity at the phylum level while driving functional differentiation at the genus level in global grassland ecosystems. Notably, genus-level LEfSe analysis (Section 2.3) further resolved niche differentiation (e.g., elevated Metarhizium in high-diversity plots), complementing phylum-level insights—this two-tiered analytical framework has been widely validated in urban forest soil studies, where Hui et al. [8] found that phylum-level analysis clarified dominant microbial trophic strategies, while genus-level analysis identified key taxa mediating plant-soil feedbacks in cold-temperate urban forests. The lack of clear phylum-level correlations for some traits (e.g., pH and Basidiomycota) reflects the functional redundancy within phyla [17]—where distinct genera may exhibit opposing responses to environmental factors (e.g., certain Basidiomycota genera thrive in acidic soils while others prefer neutral conditions), blurring phylum-level signals. This approach balances ecological generality and mechanistic detail, which is critical for disentangling complex plant-microbe-soil interactions in urban ecosystems [6,40].

Fungal communities exhibited greater pH sensitivity than bacteria (Figure 7), consistent with temperate forest fungi preferring neutral-slightly acidic habitats [45]. For example, Ascomycota abundance declined in high-pH plots (Figure 3A), potentially due to pH constraints on their lignin-degrading enzymes [39]. Xiang et al. [32] observed that elevated pH inhibits Ascomycota lignin-decomposition genes (e.g., laccases), explaining this reduction.

SWC negatively correlated with Armatimonadota (Figure 7B), suggesting this group’s adaptive decline under water-fluctuation stress [46,47]. De Vries et al. [48] noted bacterial networks are less drought-stable than fungal networks, with Armatimonadota decline potentially linked to water-stress-induced metabolic inhibition. Critically, plant species diversity indirectly elevates SOC via litter input [49], and SOC-microbial functional diversity feedbacks may be involved in enhancing nutrient cycling [37]. For instance, Verrucomicrobia’s SOC dependence may be linked to their methane oxidation and complex carbon metabolism genes [43]. Metagenomic evidence confirms that plant species diversity could potentially regulate microbial functional gene differentiation (e.g., nitrogen fixation) via root exudates, improving nitrogen cycling efficiency [50].

The PLS-PM model (Figure 9) revealed stronger direct effects of plant species diversity on fungal versus bacterial diversity. This aligns with Prober et al.’s global grassland finding that plant species diversity primarily influences fungal β-diversity [40]. The bacterial community’s dependence on soil physicochemical factors further supports Bardgett’s theory that “litter indirectly structures bacteria via pH and organic matter” [6]. Boxplot analyses showed higher fungal Shannon-Wiener indices under high diversity (Figure 5A), reflecting optimized community evenness—consistent with Lange et al.’s observation that “high diversity promotes fungal functional group evenness” [19]. Conversely, bacterial ACE index dominance at low diversity matches Dassen et al.’s finding that “oligotrophic bacteria drive richness in simplified communities” [17].

## 4. Materials and Methods

### 4.1. Study Area

Jingyuetan National Forest Park (43°52′ N, 125°21′ E) is situated approximately 18 km southeast of the city center of Changchun, Jilin Province, China [51]. Encompassing 96.38 km^2^ (water area: 5.3 km^2^), this low-mountain landscape (220–406.5 m elev.) lies in a temperate continental monsoon zone. Mean annual temperature is 4.7 °C (January: −15.1 °C; July: 23.1 °C), with annual precipitation of 645.3 mm concentrated in summer (June–August, 66.6%) [52]. The winter season extends for approximately 204 days; it includes 146 days with temperatures below 0 °C and snow depths averaging 20–25 mm [53].

### 4.2. Experimental Design

In Jingyuetan National Forest Park, Changchun City, sample plots were set up using a combination of random sampling and typical sampling methods based on the park’s topography, vegetation distribution, and ecological environment heterogeneity. Considering the variations in vegetation types, soil conditions, and topography across different areas of the park, 12 sample plots (20 m × 20 m, 400 m^2^ each) were finally established to ensure they represent the main forest stand types of the park and thereby reflect its key ecological characteristics (as shown in Table 1). To quantify plant species diversity and stratify diversity gradients, the Shannon-Wiener index (H′) was calculated for each plot based on vegetation survey data (Table 1). The plots were categorized into low (L), medium (M), and high (H) plant species diversity levels using the Jenks Natural Breaks Optimization method—a natural clustering threshold partitioning algorithm that maximizes within-group homogeneity and between-group heterogeneity. The classification thresholds were further validated by referencing the temperate urban forest diversity stratification standard [54], which defines low diversity as H′ < 0.8, medium diversity as 0.8 ≤ H′ ≤ 1.2, and high diversity as H′ > 1.2. Specifically, the low-diversity group included plots with H′ = 0.43–0.53 (*n* = 4), the medium-diversity group with H′ = 1.04–1.22 (*n* = 4), and the high-diversity group with H′ = 1.79–1.87 (*n* = 4). One-way ANOVA confirmed significant differences in Shannon-Wiener indices among the three groups (*p* < 0.01), with no overlap between groups, verifying the rationality of the gradient classification (Table 1).

The tree species composition and growth status of each plant were investigated, including tree diameter at breast height (DBH), crown width, and tree height. In each sample plot, sampling was conducted using the S-point sampling method, with 5 sampling points established randomly, soil samples were collected from the soil layer (0–20 cm) using a soil drill (inner diameter 5 cm), and soil was collected with a ring knife near the sampling points to determine soil bulk density, porosity, and water content, then, thoroughly mixing the soil samples at each sampling point. After removing sand and roots, the soil samples were divided into two parts. One part was air-dried in a cool and dry place for determining soil chemical properties, and the other part was stored at −80 °C in an ultra-low temperature refrigerator to determine soil microbial community structure and composition.

### 4.3. Determination of Soil Physicochemical Properties

Soil physicochemical properties were measured primarily according to Bao [55], with specific details as follows: soil bulk density by the ring knife method, soil water content by the oven-drying method, soil pH (water-soil ratio 2.5:1) and electrical conductivity (water-soil ratio 5:1) determined using a PHS-3E pH meter and a DDS-11A conductivity meter, respectively. Soil organic carbon was quantified by the potassium dichromate external heating method, and total phosphorus was analyzed with a continuous flow analyzer (Auto Analyzer 3; Bran + Luebbe; Hamburg, Germany) after digestion with concentrated H_2_SO_4_ and H_2_O_2_.

### 4.4. Determination of Soil Microbial

Steps such as PCR amplification with 16S rDNA specific primers, purification of amplification products, DNA sequencing, and sequence alignment were carried out to further determine the structural composition of soil microbial communities [56]. The 338F (50-ACTCCTACGGGAGGCAGCAG-30) and 806R (50-GGACTACHVGGGTWTCTAAT-30) primers were used to perform PCR amplification on the bacterial 16S rRNA, targeting the 16S V3–V4 variable region. PE reads obtained from sequencing were first spliced according to overlapping relationships, and sequence quality was checked and filtered simultaneously. The raw PE reads were first subjected to denoising to remove low-quality sequences (Q < 20). Then, OTU clustering was performed at a 97% sequence similarity threshold using UPARSE, followed by taxonomic annotation and diversity analysis. Although contemporary studies increasingly use ASVs (Amplicon Sequence Variants) for higher resolution, OTU clustering at 97% similarity is a widely accepted method for microbial community comparison in ecological studies [56]. After distinguishing samples, OTU clustering analysis and species classification analysis were performed. Based on OTUs, various diversity indices can be analyzed. Based on the results of OTU clustering analysis, various diversity indices can be analyzed on OTUs, and sequencing depth can be checked. Based on taxonomic information, statistical analysis of community structures at different taxonomic levels can be performed. Based on the above analyses, a series of in-depth statistical and visualization analyses, such as multivariate analysis and difference significance testing, can be conducted on the community composition and phylogenetic information of samples. The above microbiological and bacteriological tests were carried out on the Major bio platform.

### 4.5. Data Analysis

Plant species diversity was quantified using the Shannon-Wiener index (*H′*), which integrates species richness and evenness to comprehensively characterize community diversity [57]. The index is calculated as:

$$H^{\prime }=-\sum_{i=1}^{S}\left(p_{i}\ln p_{i}\right)$$

where *S* is the total number of species, *p_i_* is the proportion of individuals belonging to the *i*-th species (*p_i_ = n_i_*/*N*), *n_i_* is the abundance of the *i*-th species, and *N* is the total abundance of all species.

Subsequently, Simpson’s diversity index (*D*), which integrates species richness and dominance to characterize microbial community diversity, was calculated as:

$$D=1-\sum_{i=1}^{S}p_{i}^{2}$$

Here, *S* is the total number of microbial species (or Operational Taxonomic Units, OTUs), and *p_i_* is the proportion of individuals belonging to the *i*-th species *p_i_ = n_i_*/*N*, where *n_i_* is the abundance of the *i*-th species and *N* is the total abundance of all species). This index ranges from 0 to 1, with values approaching 1 indicating higher diversity (i.e., lower dominance and more uniform species abundance distribution) in the microbial community.

Subsequently, Pielou’s evenness index (*J*′), which reflects the uniformity of individual distribution across species, was derived as:

$$J^{\prime }=\frac{H^{\prime }}{\ln S}$$

Here, *H*′ is the Shannon-Wiener diversity index and *S* is the total number of species. This index ranges from 0 to 1, with values approaching 1 indicating a more uniform distribution of individuals among species.

To comprehensively characterize species richness, especially for communities with numerous rare species (e.g., microbial communities), the Abundance-based Coverage Estimator (*ACE*) was employed to estimate the total species richness by correcting for rare taxa.

$$C=1-\frac{n_{1}}{N}$$

$$ACE=S_{abund}+\frac{S_{rare}}{C}+\frac{\left(\mathrm{var}-1\right)\times S_{rare}}{C^{2}}$$

In this formula, *S_abund_* denotes the number of abundant species (with occurrence frequency ≥ 10), *S_rare_* represents the number of rare species (with occurrence frequency ≤ 9), *C* is the coverage calculated as *C =* 1 *− n*_1_/*N* (where *n*_1_ is the number of individuals belonging to species that occur only once, and *N* is the total number of individuals across all species), and var is the variance of the abundances of rare species. This index is particularly valuable for minimizing underestimation caused by rare species, thus providing a more accurate characterization of species richness in complex communities.

Statistical analyses were performed using IBM SPSS Statistics 26 (IBM Corp., Armonk, NY, USA). Prior to one-way ANOVA, the Shapiro-Wilk test was used to verify the normality of soil physicochemical and microbial community datasets, and Levene’s test was conducted to assess homogeneity of variances. All datasets met the assumptions of normality (*p* > 0.05) and homoscedasticity (*p* > 0.05), supporting the use of parametric ANOVA. One-way analysis of variance (ANOVA) was applied to assess differences in microbial community parameters across plant species diversity levels. The post hoc comparisons were conducted using Fisher’s Least Significant Difference (LSD) test at α = 0.05 level. Fisher’s least significant difference (LSD) test was selected for post-hoc multiple comparisons because it is robust for balanced experimental designs (*n* = 4 per diversity group) and enables precise quantification of pairwise differences in soil traits and microbial diversity indices—critical for identifying gradient-specific effects of plant diversity. For data visualization, boxplots were generated using Origin 2021 (Origin Lab Corp., Northampton, MA, USA). The partial least squares path modeling (PLS-PM) analysis, aimed at exploring the relationships between plant species diversity and microbial community characteristics, was conducted in R4.3.2 (R Foundation for Statistical Computing, Vienna, Austria) using the “plspm” package. For microbial OTU composition visualization, Venn diagrams were generated by calculating the intersection and uniqueness of OTUs across plant diversity gradients using the “VennDiagram” package in R4.3.2, with OTU clustering performed at a 97% sequence similarity threshold via UPARSE. Soil microbial community composition bar plots (phylum/genus level) were constructed by normalizing OTU abundances to relative values (percentage of total OTUs) in each sample, and intergroup differences in dominant genus abundances were tested via Welch’s t-test with Benjamini-Hochberg false discovery rate (FDR) correction (α = 0.05). Fungal functional guild (FUNGuild) and bacterial COG functional annotation analyses were conducted by mapping OTU sequences to corresponding databases, with functional group abundances quantified as relative proportions; differences in functional group composition across diversity gradients were assessed via Kruskal-Wallis H-test. Correlation heatmaps between soil physicochemical properties and microbial communities were generated using the “corrplot” package in R4.3.2, with correlation coefficients calculated via Spearman’s rank correlation (for non-normal data) and significance determined via two-tailed *t*-tests (*p* < 0.05, *p* < 0.01, *p* < 0.001). Mantel test heatmaps were constructed in R4.3.2 using the “vegan” package, with Bray-Curtis dissimilarity used to quantify microbial community compositional differences, and Mantel statistics (r) and significance (*p*) calculated via 999 permutations to assess the strength of associations between microbial communities and soil physicochemical factors.

## 5. Conclusions

In conclusion, this study systematically elucidates the synergistic mechanisms among plant species diversity, soil microbial communities, and physicochemical properties in urban forest parks. High plant species diversity significantly drives microbial functional sorting: fungal communities in high-diversity plots contain 43.55% unique OTUs with Ascomycota abundance reaching 39.34%, while bacterial communities are dominated by Actinobacteriota (42.32%) and Proteobacteria (28.15%), indicating litter heterogeneity and root exudate diversity as core drivers of microbial assembly. Additionally, soil organic carbon (SOC) positively correlates with Verrucomicrobia, Acidobacteriota abundance increases with decreasing SOC (highlighting carbon source-driven microbial differentiation), and fungal communities are more sensitive to pH changes than bacteria (with reduced Ascomycota in high-pH plots potentially linked to inhibited lignin-degrading enzyme activity).

Furthermore, plant species diversity indirectly increases SOC via litter input and activates functional genes of nitrogen-fixing (e.g., *Rhizobium*) and phosphorus-solubilizing bacteria, significantly enhancing soil nutrient cycling efficiency. Notably, mixed plantings (arbor-shrub-herb) are likely to promote microbial functional network stability by optimizing microhabitat heterogeneity and resource diversity, which may help improve urban forest ecological services such as carbon sequestration, nutrient provision, and air purification.

This study provides a scientific basis for biodiversity configuration and soil health maintenance in urban forest management, underscoring the critical role of plant-microbe-soil interactions in sustaining urban ecosystem functions.

It should be noted that this study has certain limitations in terms of sample size: only 12 plots (4 replicates per diversity gradient) were established, and the pooling of soil cores within plots resulted in *n* = 4 per treatment for statistical analysis. Given the high spatial heterogeneity of urban forests, this may limit the broad generalizability of the conclusions and the ability to detect subtle ecological variations [8]. However, this sampling design is consistent with published studies on urban soil microbiomes [9], which have verified that moderate replication with strict habitat standardization (e.g., uniform elevation and soil parent material in this study) can effectively capture core ecological patterns. In addition, the large effect sizes of key indicators (e.g., 48.4% higher SOC in high-diversity plots, 43.55% unique fungal OTUs in high-diversity plots) have significant biological meaning, which can fully support the core conclusions of this study. Future research can expand the sampling scope to multiple temperate urban forests and combine controlled experiments to verify the causal relationship between plant diversity and microbial communities, further improving the generalizability of the conclusions.

## Figures and Tables

**Table 1:** Shannon-Wiener index, dominant, and subdominant species across plots with low (L), medium (M), and high (H) plant species diversity.

Level	Shannon-Wiener Index	Dominant Species	Subdominant Species	Number of Plants	Species Richness
L	0.43	*Larix olgensis* A. Henry	*-*	29	4
	0.47	*Quercus mongolica* Fisch. ex Ledeb.	*-*	21	3
	0.50	*Quercus mongolica* Fisch. ex Ledeb.	*-*	23	4
	0.53	*Pinus sylvestris var. mongolica* Litv.	*-*	52	5
M	1.04	*Pinus sylvestris var. mongolica* Litv.	*-*	22	4
	1.05	*Pinus tabuliformis var. mukdensis* Uyeki	*-*	31	4
	1.15	*Pinus koraiensis* Siebold & Zucc.	*-*	28	6
	1.22	*Pinus sylvestris var. mongolica* Litv.	*Pinus koraiensis* Siebold & Zucc.	14	5
H	1.79	*Pinus sylvestris var. mongolica* Litv.	*Acer mono* Maxim.	43	12
	1.82	*Pinus sylvestris var. mongolica* Litv.	*Acer mono* Maxim.	46	9
	1.82	*Quercus mongolica* Fisch. ex Ledeb.	*Tilia mandshurica* Rupr. & Maxim.	54	9
	1.87	*Pinus koraiensis* Siebold & Zucc.	*Ulmus pumila* L.	31	9

## Data Availability

The research data supporting the conclusions of this study are not publicly available at present. This is because the dataset will be utilized for subsequent extended research work, so as to ensure the continuity of the relevant research and the consistency of in-depth data analysis. For legitimate academic inquiries that comply with ethical and academic norms, interested parties may contact the corresponding author (Dan Zhang, zhangdan@ccu.edu.cn) after the completion of the follow-up research, to request access to the data upon appropriate review and approval.

## Abbreviations

The following abbreviations are used in this manuscript:

ACE	Abundance-based Coverage Estimator
COG	Clusters of Orthologous Groups of proteins
DBH	Diameter at Breast Height
EC	Electrical Conductivity
FUNGuild	Fungal Functional Guild
LSD	Least Significant Difference
OTUs	Operational Taxonomic Units
PLS-PM	Partial Least Squares Path Modeling
PE	Paired-End
16S rDNA	16S Ribosomal Deoxyribonucleic Acid
SBD	Soil Bulk Density
SOC	Soil Organic Carbon
SOM	Soil Organic Matter
SWC	Soil Water Content
TP	Total Phosphorus
